# Ethylene-Mediated Modulation of Bud Phenology, Cold Hardiness, and Hormone Biosynthesis in Peach (*Prunus persica*)

**DOI:** 10.3390/plants10071266

**Published:** 2021-06-22

**Authors:** Jianyang Liu, Md Tabibul Islam, Sangeeta Sapkota, Pratibha Ravindran, Prakash P. Kumar, Timothy S. Artlip, Sherif M. Sherif

**Affiliations:** 1 Alson H. Smith Jr. Agricultural Research and Extension Center, Virginia Tech, School of Plant and Environmental Sciences, Winchester, VA 22602, USA; liujy4@vt.edu (J.L.); tabibul@vt.edu (M.T.I.); sangee7@vt.edu (S.S.); 2 Department of Biological Sciences, National University of Singapore, Singapore 117543, Singapore; pratibha@nus.edu.sg (P.R.); prakash.kumar@nus.edu.sg (P.P.K.); 3 Apple Biotechnology, USDA-ARS-Appalachian Fruit Research Station, Kearneysville, WV 25430, USA; tim.artlip@usda.gov

**Keywords:** peach (*Prunus persica*), bud dormancy, ethylene, spring frost, plant hormones

## Abstract

Spring frosts exacerbated by global climate change have become a constant threat to temperate fruit production. Delaying the bloom date by plant growth regulators (PGRs) has been proposed as a practical frost avoidance strategy. Ethephon is an ethylene-releasing PGR found to delay bloom in several fruit species, yet its use is often coupled with harmful effects, limiting its applicability in commercial tree fruit production. Little information is available regarding the mechanisms by which ethephon influences blooming and bud dormancy. This study investigated the effects of fall-applied ethephon on bud phenology, cold hardiness, and hormonal balance throughout the bud dormancy cycle in peach. Our findings concluded that ethephon could alter several significant aspects of peach bud physiology, including accelerated leaf fall, extended chilling accumulation period, increased heat requirements, improved cold hardiness, and delayed bloom date. Ethephon effects on these traits were primarily dependent on its concentration and application timing, with a high concentration (500 ppm) and an early application timing (10% leaf fall) being the most effective. Endogenous ethylene levels were induced significantly in the buds when ethephon was applied at 10% versus 90% leaf fall, indicating that leaves are essential for ethephon uptake. The hormonal analysis of buds at regular intervals of chilling hours (CH) and growing degree hours (GDH) also indicated that ethephon might exert its effects through an abscisic acid (ABA)-independent way in dormant buds. Instead, our data signifies the role of jasmonic acid (JA) in mediating budburst and bloom in peach, which also appears to be influenced by ethephon treatment. Overall, this research presents a new perspective in interpreting horticultural traits in the light of biochemical and molecular data and sheds light on the potential role of JA in bud dormancy, which deserves further attention in future studies that aim at mitigating spring frosts.

## 1. Introduction

Temperate fruit production is often challenged by late spring frosts; the sporadic freezing temperatures that occur during spring after the weather begins to warm up. Spring frosts can damage or kill newly developed flowers and fruitlets, thereby substantially reducing fruit yield and quality. The concerns of spring frosts have been rising in recent years, especially in the context of climate change and its effects on the phenology of woody species and the frequency of freezing events during springtime [1,2,3]. Spring frosts impact a wide range of temperate perennials and threaten the production of several economically important fruit species such as stone fruits. Stone fruits belong to the genus *Prunus* in the *Rosaceae* family, and include among its members peaches, sweet cherries (*P*. *avium*), and almonds (*P*. *dulcis*). In temperate regions, the flowering time of stone fruits often coincides with the occurrence of spring frosts, making floral organs especially prone to frost injuries. 

The current approaches in frost management can be active or passive [4]. Active approaches alter the microclimate of the orchards by using devices such as wind machines, surface irrigation, heaters, etc. These techniques produce immediate and direct effects, but are generally cost-ineffective, relatively unreliable, and environmentally unsustainable. Passive methods, on the other hand, are mostly preventive and preemptive. These include cultivation of cold-hardy or late-bloom cultivars, proper selection of planting site, and application of plant growth regulators (PGR) to enhance the freezing tolerance [5,6] or delay flowering date to avoid potential freezes [7,8]. The use of PGRs, e.g., ethephon, is gaining more popularity owing to its ease of use and cost-effectiveness.

Ethephon (2-chloroethylphosphonic acid) is an ethylene-based PGR that has demonstrated effectiveness in protecting fruit trees against spring frosts. Ethephon decomposes to ethylene after entering plant cells, and its effects are generally attributed to ethylene [9]. Many studies have documented that fall applications of ethephon can delay bloom in many fruit species, especially stone fruits. In peach, for instance, ethephon can delay bloom for up to 18 days post the natural bloom date [10], which may greatly reduce the odds of frost damage. However, the efficacy of ethephon largely depends on concentrations and application timing. Application timing is specifically critical, as harmful effects on flower buds were observed when the application was made after endodormancy [11], and effects on bloom time became insignificant when ethephon was applied during endodormancy [7]. In addition to delaying bloom, ethephon was also found to enhance flower bud hardiness in late winter by delaying deacclimation [11]. However, the practical benefits of ethephon are largely offset by its harmful effects, which include gummosis, leaf yellowing and abscission, terminal dieback, flower abscission, floral bud failure, and reduced fruit yield [12,13,14]. These detrimental effects have posed a great limitation in using ethephon for bloom delay in commercial production settings. Exploring chemical alternatives to induce bloom delay without risking tree health and yield would require a better understanding of the mechanisms by which ethephon influences flowering, which is largely lacking.

To survive the freezing temperatures in wintertime, the buds of deciduous perennial species enter a phase of dormancy that is characterized by the lack of visible growth or primordia activity. Based on the source of repression signals, winter dormancy can be divided into two sequential phases: endodormancy and ecodormancy. In endodormancy, the growth of terminal and lateral buds is repressed by intrinsic factors, and buds become unresponsive to favorable growth conditions; whereas, in ecodormancy, growth is solely inhibited by unfavorable environmental conditions [15]. During endodormancy, temperate plants require a certain number of chill hours, known as chilling requirement (CR) before the buds can overcome endodormancy and transition to ecodormancy. After endodormancy completion, a period of warm temperatures is required to satisfy heat requirement (HR) for proper bud break and bloom. Both chilling and heat requirements are genetically controlled and are highly species- and cultivar-specific [16]. Commonly, CR is quantified by using temperature-based models, such as the chilling hour (CH) model [17], Utah model [18], and dynamic model [8]. The CH model assumes that only temperatures between 0 and 7.2 °C are effective in satisfying CR, and each hour within this temperature range registers as one chilling hour. The Utah model and dynamic model take into consideration the effects of warm weather on accumulated chill, and are suitable for regions where warm spells during dormancy are a concern. The dynamic model assumes a two-step biochemical mechanism for endodormancy release and calculates chilling accumulation as chilling portion (CP) [19]. Heat requirement is commonly estimated as the growing degree hours (GDH) that accumulate from the release of endodormancy to the flowering date (when 50% of flowers are open, F_50_) [20]. 

The phenology of dormancy and blooming is tightly controlled by a complex orchestration of several mechanisms, in which phytohormones play a crucial role [21,22]. In this study, we hypothesized that ethephon may alter blooming of peach by modulating the metabolisms of phytohormones during dormancy. Specifically, in the present study, we aimed to (1) comprehensively evaluate the overall effects of ethephon on the physiology of peach cultivars during dormancy-flowering stage, with an emphasis on chilling and heat requirements, and blooming; (2) elucidate the dynamics of major phytohormones during dormancy and as affected by ethephon applications; and (3) examine the transcriptional regulation of phytohormones by analyzing the expression of genes responsible for their metabolisms. In this study, we used two peach cultivars to address these research objectives, with an attempt to tap into the potential of using ethephon as a frost mitigation tool, while keeping its negative impacts at tolerable levels.

## 2. Materials and Methods 

### 2.1. Location and Plant Materials

This study was conducted at the Alson H. Smith JR Agricultural Research and Extension Center (AREC), Winchester, VA, the United States (39.11, −78.28). This area features a humid subtropical type of climate (Köppen climate classification Cfa), with average temperatures at the peak of hot and cold seasons being 30 °C and −3.3 °C in July and January, respectively. The two peach cultivars used in this study were ‘Redhaven’ and ‘Sunhigh’, which were planted in 2012 and 2015, respectively, and both grafted to ‘Lovell’ rootstock. In this study, we used ‘Redhaven’ as the primary plant material for the assessment of ethephon effects on leaf abscission, cold hardiness, and hormonal levels, as this cultivar is widely planted and highly prized worldwide.

### 2.2. Experimental Design and Treatments 

Two field trials were conducted in fall of 2019 to examine the effects of different concentrations and application times of ethephon on peach. The concentration effects of ethephon were tested on ‘Redhaven’ using a randomized complete block design (RCBD), in which each of the three rows was treated as a block. In each block, three ethephon concentrations of 100, 300, and 500 ppm (referred to as ET-100, -300, and -500 ppm thereafter) plus a control were randomly assigned to four groups, each with two adjacent trees; ethephon was applied on 24 October 2019, corresponding to 50% of leaf fall (LF). In the ‘Sunhigh’ orchard, complete randomization design (CRD) was used to examine the effects of application timing. Of the four randomly selected groups, each having three adjacent trees, one was designated as untreated control and the other three were treated with 500 ppm ethephon on 1 October 2019 and 9 November 2019, which correspond to 10, 50, and 100% LF stages, respectively. In both experiments, at least two buffer trees were included between different treatment trees to reduce the effects of spray drift. Upon application, Motivate (Fine American Inc., Walnut Creek, CA, USA) containing 21% ethephon was diluted to the desired concentration and mixed with a nonionic surfactant, Regulaid (Kalo Inc., Overland Park, KS, USA), at 250 ppm. For each treatment, an air blast sprayer was used to spray both sides of the trees, and the controls were sprayed with the surfactant only.

### 2.3. Chilling and Heat Requirements

Chilling and heat accumulation were computed from meteorological data obtained from each orchard. Data loggers (EasyLog, Lascar) were kept in enclosed wooden shelters 1.2 m above the ground and recorded temperatures at 10 min intervals. Experiments in this study were scheduled based on the accumulation of CH, and the corresponding CP was also provided. The CH accumulation was calculated using the 0–7.2 °C model [17], in which the hourly temperatures within the range of 0–7.2 °C registered as 1 chilling hour (CH), and temperatures outside of this range were not considered. The CP was calculated according to Fishman et al. [19]. Starting at 500 CH, three one-year old branches, each with at least 15 floral buds, were collected weekly from each treatment. After removal of the terminal buds, the branches were placed in a beaker with the basal ends 3 cm in a floral solution prepared with Floralife (Smithers-Oasis Co., Walterboro, SC, USA), which was replaced weekly; the basal ends were trimmed back for 1 cm weekly to avoid infections. The branches were kept in a growth chamber with conditions favoring bud growth (photoperiod with fluorescent lamps at 135 μmol·m^−2^·s^−1^ of 16 h at 25 °C, a dark period of 8 h at 20 °C, and a constant relative humidity of 65%). Budbreak was defined when the buds reached the calyx green stage after 14 days in the growth chamber, and CR corresponds to the CH when budbreak rate reached 50%.

Heat accumulation was computed as growing degree hours (GDH), according to the method proposed by Anderson et al. [9]. In this model, hourly temperatures less than 4.5 °C registered no GDH; temperatures in the interval of 4.5–25 °C were adjusted by subtracting 4.5 °C; and temperatures greater than 25 °C were registered as 20.5 (25–4.5) °C. Total GDH was calculated by summing all the GDH from the time when GDH first became detectable to the predetermined ending time. The first GDH in 2020 was recorded on 21 January. Heat requirements (HR) were calculated as the GDH accumulated from the release of endodormancy to the flowering date F_50_ (50% of opened flowers).

### 2.4. Leaf Abscission Assessments

The effect of ethephon in causing leaf abscission was evaluated in the ‘Redhaven’ orchard 7 days after ethephon applications. Ten branches were randomly selected per tree and ten leaf nodes starting from the branch tip were recorded as attached or fallen. Leaf abscission rate was calculated as the ratio of number of leafless nodes to the total leaf nodes. 

### 2.5. Endogenous Ethylene Levels in Leaves and Buds after Ethephon Application

To evaluate ethephon effects on ethylene induction, we quantified ethylene production rate in buds and leaves of ‘Redhaven’ at eight time points (1, 2, 3, 6, 9, 15, 21, and 30 days) after treatment with ethephon (500 ppm) at 10 and 90% leaf fall stages. At each time point, about 15 buds and 25 g of leaves (fresh weight) were sampled and placed in sealed tubes (5 mL) and jars (3750 mL), respectively. Samples were maintained in a growth chamber with a 16:8 h light:dark cycle at 22 °C for 24 h before quantification. At analysis, 1 mL of air from the headspace of each container was withdrawn with a syringe and manually injected into a gas chromatograph (Agilent 7890 A, Agilent Technologies, Wilmington, DE, USA) equipped with a flame ionization detector (FID). The concentration of ethylene (ppm) measured by the gas chromatography was converted to nmol·g^−1^·h^−1^ by factoring container size, dry weight (DW), and the duration of evolution. 

### 2.6. Evaluation of Bloom Progression

The progression of flower development was recorded by periodically counting the number of open blossoms in each orchard. At the pink-bud stage, four branches from each tree were randomly selected, with each having at least 20 floral buds, which were counted as the initial bud number. Number of open flowers (anthers visible) were counted every 1–3 days until all buds entered the full bloom stage (100% of flowers are open). Blooming rate was calculated as the ratio of open blossoms to the initial bud number of buds per tree, and the flowering date (F_50_) for each treatment was determined when the blooming rate reached 50%.

### 2.7. Evaluation of Fruit Set, Fruit Size and Tree Injury 

In the ‘Redhaven’ and ‘Sunhigh’ orchards, the numbers of fruits on the marked branches were counted 2, 4, and 6 weeks after full bloom. Fruit set (%) was calculated as the number of fruit set divided by the number of flowers times 100. Fruit size was evaluated in the ‘Redhaven’ and ‘Sunhigh’ orchards at 2, 4 and 6 weeks after full bloom (WAFB). A total of 10 fruits were randomly selected from each tree and the diameter of each was recorded by measuring the widest part of each fruit using a digital caliper. 

To evaluate the magnitude of damage induced by ethephon treatments, we collected and weighed the dead branches (mostly 1- and 2-year-old branches) from each experimental tree of ‘Redhaven’ and ‘Sunhigh’ 6 WAFB (Figure S1). 

### 2.8. Determination of Bud Cold Hardiness 

The cold hardiness of the peach buds was estimated using the differential thermal analysis (DTA). In this analysis, the buds are subject to progressive freezing until the symplastic water in the bud tissues freezes to release the heat of fusion, which is recorded as low temperature exotherm (LTE). The cold hardiness of the ‘Redhaven’ buds was examined on the control and ethephon-500 ppm trees at 200, 600, 1000, 1250, and 1400 CH (16.4, 33.8, 56.2, 68.4, and 74.6 CP, respectively). In this assessment, three one-year-old shoots, each from a replicate tree of control and ethephon treatments were harvested from the ‘Redhaven’ orchard and kept on ice until processed. Flower buds were excised from shoots with a small portion of subtending shoot tissue attached. One cut end of each bud was coated with ice nucleation active (INA) bacteria to ensure freezing of the bulk water [23]. One or two buds from each treatment were placed onto each of the nine sample cells, which are arranged on a sample tray; each cell was covered by a tight-fitting foam (Brock University Electronics Shop, Brock University, St. Catherines, ON, Canada) (Figure S2A,B). The trays were then placed in the Tenney Junior Environmental Test Chamber, Model TJR, with a Watlow F4 controller (Thermal Product Solutions, New Columbia, PA, USA) (Figure S2C). Data were acquired by a Keithley 2700 Multimeter/Data Acquisition System (Keithley, Cleveland, OH, USA), which was driven by the Bud Freezer software (v 1.2, Brock University). The temperature profile of the environmental chamber was programmed as follows: equilibration for 1 h at 4 °C, ramp to −35 °C in −2 °C/h decrements, soak 3 h at −35 °C, ramp to 4 °C with 5 °C/h increments, hold at 4 °C. The voltage signal from each bud sample was recorded at a 1 s interval as the temperature was progressively lowered and LTE was detected as a peak when the symplastic water in the tested buds started to freeze (Figure S2D).

### 2.9. Quantification of Phytohormones 

Plant hormones were analyzed using a high-performance liquid chromatography coupled with a mass spectrometry (LC–MS) system. To prepare for hormone quantification and gene expression analysis (see next section), dormant floral buds of ‘Redhaven’ were sampled at eight time points based on CH accumulation of 20, 200, 400, 600, 800, and 1000 (0.6, 16.4, 27.6, 33.8, 48.9, and 56.2 CP, respectively) and GDH accumulation of 1000 and 3000, which correspond to calendar dates of 14 October, 11 November, 27 November, 9 December, 27 December of 2019, and 15 January, 14 February, and 12 March of 2020, respectively. Collected buds were immediately frozen in liquid nitrogen and kept at −80 °C until further processing. Bud samples from each replicate were homogenized in liquid nitrogen using a Geno/Grinder. About 100 mg ground sample was weighed and mixed with 5 times (*w/v*) 80% ice-cold HPLC-grade methanol, vortexed for 1 min, and kept at 4 °C in the dark for 3 h. Samples were centrifuged at 14,000 rpm at 4 °C for 20 min, and the supernatants were centrifuged at 4 °C for another 10 min to further remove the remaining debris. The supernatants were completely dried with a SpeedVac and resuspended in 100 µL of 80% methanol. Samples were injected into the ZORBAX Eclipse Plus C18 column (mobile phase A: water containing 0.1% acetic acid; mobile phase B: acetonitrile containing 0.1% acetic acid) coupled to an Agilent 6490 Triple Quadrupole LC–MS System with AJS technology. Quantification of GA9, ABA, JA, and JA-Ile was performed as described by Seo et al. [24]. 

### 2.10. RNA Extraction and Gene Expression Analyses

Total RNA was extracted from bud samples using a CTAB method previously described by Sherif et al. [25]. All RNA extracts were DNase treated and purified using the RNA clean and concentrator kit (Zymo Research, Irvine, CA, USA). For cDNA synthesis, 2 μg of purified RNAs was reverse transcribed using the cDNA Synthesis Kit (Applied Biosystem, Foster City, CA, USA) according to the manufacturer’s instructions. The quantitative real-time PCR (qPCR) reactions were performed using the CFX Connect Real-Time System (Bio-Rad, Hercules, CA, USA) and the SsoFast EvaGreen Supermix (BioRad, USA). The primers were designed using Primer3Plus (https://www.bioinformatics.nl/cgi-bin/primer3plus/primer3plus.cgi, accessed on 24 March 2020) to target the key genes in the biosynthesis pathways of plant hormones ABA, which include two 9-cis-epoxycarotenoid dioxygenase genes (*NCED2* and *NCED3*); GA, including two GA 20-oxidases (*GA20-OX1* and *GA20-OX2*); ethylene, including ACC-synthase (*ACS*) and ACC-oxidase (*ACO*); and JA, including linoleate 13S-lipoxygenase (*13-LOX*), allene oxide synthase (*AOS*), allene oxide cyclase (*AOC*), OPDA-reductase 3 (*OPR3*), and JA-amino synthetase (*JAR*) (Table S1). The *β*-*Actin* gene (Prupe.6G163400.1) was used as the endogenous control for gene expression normalization. The expression of each gene was calculated relative to the gene expression of the control sample (20 CH), using the CFX manager software (Bio-Rad). The normalized relative expression data represent the mean (±) standard error (SE) of three biological replicates, each including three technical replicates. 

### 2.11. Data Analyses

The cold hardiness data were processed by the Bud Processor software (v1.0, Brock University) that allows visualization of temperature (independent variable) versus voltage (dependent variable). The low temperature exotherms (LTEs) were then identified and stored for statistical analysis by the Bud LTE software (v 1.0, Brock University). The Bud LTE software presented the data for the mean ± standard deviation of LTE10, LTE50, and LTE90, which denote the temperatures at which 10, 50, and 90% of the buds are killed, respectively. In particular, LTE50 corresponds to the cold hardiness (Hc) [26].

All statistical analyses in this study were performed using R version 3.6.3 (R Core Team, 2020). One-way ANOVA was conducted for each experiment to test the ethephon effect on the metrics of interest; Tukey HSD was used for multiple comparisons of significant effects from ANOVA models (*p* < 0.05).

## 3. Results 

### 3.1. Accumulation of CH and GDH

**Figure 1:**
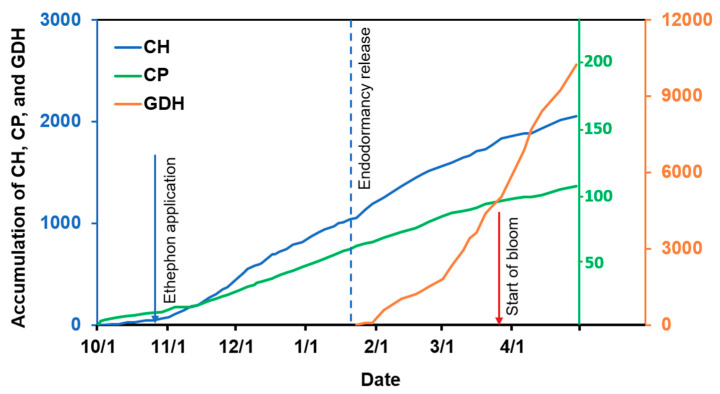
Accumulation of chilling hours (CH), chilling portions (CP), and growing degree hours (GDH) in the ‘Redhaven’ orchard. Major events are indicated with arrows. CH accumulation began on 7 October 2019, ethephon was applied on 24 October 2019, endodormancy of the control ended on 22 January 2020, GDH accumulation began on 23 January 2020, flowering of the control began on 18 March.

Temperature data recorded in the ‘Redhaven’ orchard were used to show the cumulative CH and GDH and several major events in this study (Figure 1). CH accumulation began on 7 October 2019 and continued to increase progressively thereafter, showing a pattern similar to that of chilling portion (CP) based on the dynamic model. Ethephon was applied at 50% leaf fall (LF) which corresponded to 40 CH. Chilling requirement for the control of ‘Redhaven’ was fulfilled at 1046 CH. The GDH accumulation started on 23 January 2020 and increased rapidly, reaching approximately 4500 on 18 March, when the control started blooming.

### 3.2. Ethephon Accelerated Leaf Abscission 

**Figure 2:**
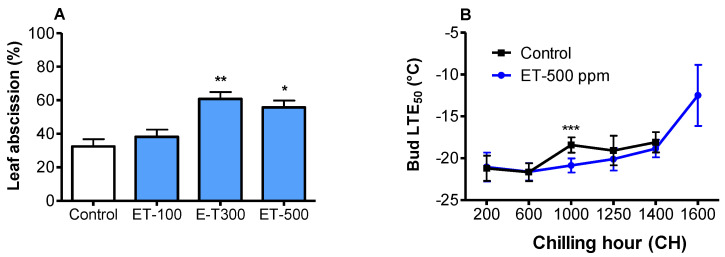
Effects of ethephon (ET) on leaf abscission and cold hardiness of ‘Redhaven’. (**A**) Leaf abscission 7 days after ethephon applications. Bars represent the mean ± SE of three biological replicates (10 branches/replicate); (**B**) LTE_50_ (temperature at which 50% of the buds are killed) of flower buds during endodormancy and ecodormancy. Each point represents the mean LTE_50_ ± SE of 12–18 individual buds. Asterisks *, **, and *** indicate significance at *p* < 0.05, 0.01, and 0.001, respectively. CH accumulation corresponds to calendar dates of 15 November and 11 December of 2019, and 5 January, 6 February, 14 February, and 4 March of 2020, respectively.

The induction of leaf abscission has been linked to the use of ethephon. The defoliation effects of ethephon were evaluated on ‘Redhaven’ one week after application. The results showed that ET-300 and -500 ppm treatments induced 60.1% (± 4.8) and 55.7% (± 4.2) of leaf abscission, respectively (Figure 2A), nearly two times more than the control (*p* < 0.01). In contrast, no significant defoliation was observed in the ET-100 ppm treatment, indicating that ethephon effects on tree defoliation is concentration-dependent.

### 3.3. Ethephon Improved Cold Hardiness 

The ethephon effects on the cold hardiness of dormant buds were evaluated by computing the LTE_50_ (the freezing temperatures at which 50% of the flower buds are killed) of the control and the ET-500 ppm treatment (Figure 2B). The LTE_50_ was well below −20 °C for both the control and ET treatment at 200 and 600 CH, and then increased gradually starting at 1000 CH, at which the LTE_50_ of ET treatment was significantly lower than that of the control (*p* < 0.001). At 1600 CH, the ET treatment lost 9.1 °C of cold hardiness, compared to its maximum cold hardiness at 600 CH (−21.6 °C), whereas the LTE_50_ of the control buds was undetectable, indicating a complete loss of cold hardiness at this point. 

### 3.4. Ethylene Production in Leaves and Buds

**Figure 3:**
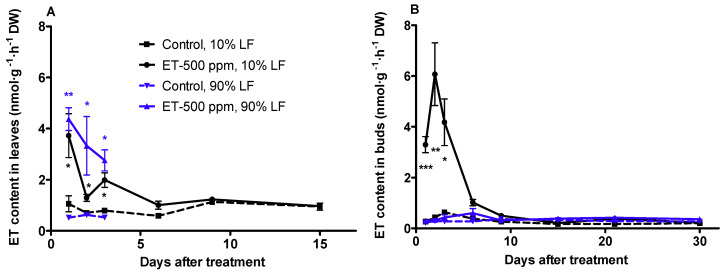
Dynamics of ethylene levels in leaves (**A**) and buds (**B**) of ‘Redhaven’ in response to ethephon application of 500 ppm at 10 and 90% LF. Ethylene levels in leaves were measured until the complete abscission. Each point represents the mean ± SE of size *n* = 3. Asterisks *, **, and *** indicate significant difference between ET treatments and the control at *p* < 0.05, 0.01, and 0.001, respectively. ET, ethephon; LF, leaf fall; DW, dry weight.

Leaves are believed to be the primary organs that convert exogenously applied ethephon to ethylene [27,28]. Our results indicated that ethylene production in leaves after ethephon applications at 10 and 90% LF reached its maximum level of 3.7 and 4.4 nmol·g^−1^·h^−1^ one day after application, both of which are greater than the respective controls (Figure 3A). The peak rates of ethylene production were followed by a rapid decline in both treatments. During the first three days after application, there was no significant difference in ethylene production between 10 and 90% LF treatments, but they were all significantly higher than the control. Ethylene production in the 10% LF treatment decreased to 1.2 nmol·g^−1^·h^−1^ six days after application and remained low thereafter. Due to accelerated leaf abscission, leaves became unavailable 15 and 3 days after ethephon application at 10 and 90% LF, respectively.

We also measured the rate of ethylene production in flower buds up to 30 days after ethephon application, when no difference between treatment and the control could be detected. When ethephon was applied at the 10% LF, ethylene production in buds peaked two days after application, reaching 6.07 nmol·g^−1^·h^−1^, approximately 14 times higher than the control (Figure 3B). After this peak, ethylene levels decreased quickly and returned to the control level nine days after application. In contrast to the high ethylene production in the 10% LF treatment, ethylene levels in the 90% LF application remained extremely low, approximating the levels of the control during the course of the experiment.

### 3.5. Ethephon Increased Chilling and Heat Requirements 

The effects of ethephon on chilling requirements was assessed by recording the CH and CP that were needed for 50% of the floral buds to break dormancy under forcing conditions (Table 1). In ‘Redhaven’, the floral buds of ethephon treatments generally required more chilling than the control to break endodormancy, with ET-500 ppm requiring 131 more CH (5.6 CP) than the control (*p* < 0.05) to achieve cold requirements. Though the ethephon treatments of 100 and 300 ppm also increased the CR when compared to the control, the differences were not significant. Similar increase of CR was also observed in ‘Sunhigh’, in which the CR of the three ethephon treatments were all significantly higher than that of the control, with CR increases ranging from 287 to 340 CH (16.2 to 21.5 CP), and no significant differences among the ethephon treatments. 

Heat requirement (HR) was calculated as the accumulation of GDH from the date when the CR was fulfilled in each treatment to the date when 50% of flower buds were in bloom (Table 2). In ‘Redhaven’, the ethephon treatments exhibited concentration-dependent effects on increasing HR, in which the three concentrations of 100, 300, and 500 ppm extended HR by 245, 578, and 901 GDH, respectively, with HR of the ET-500 ppm treatment being significantly higher (*p* < 0.05) than the control, but no significant differences were found among ethephon treatments. Similarly, significant HR increases were found in all the three ethephon-treated ‘Sunhigh’ trees (*p* < 0.05). HR increases appeared to depend on the level of leaf fall (LF), with ET-10% LF showing the highest HR (882 GDH), followed by ET-50% (696 GDH), and ET-100 % (583 GDH), but there was no significant difference among ethephon treatments. 

### 3.6. Ethephon Delayed Bloom in Peach

**Figure 4:**
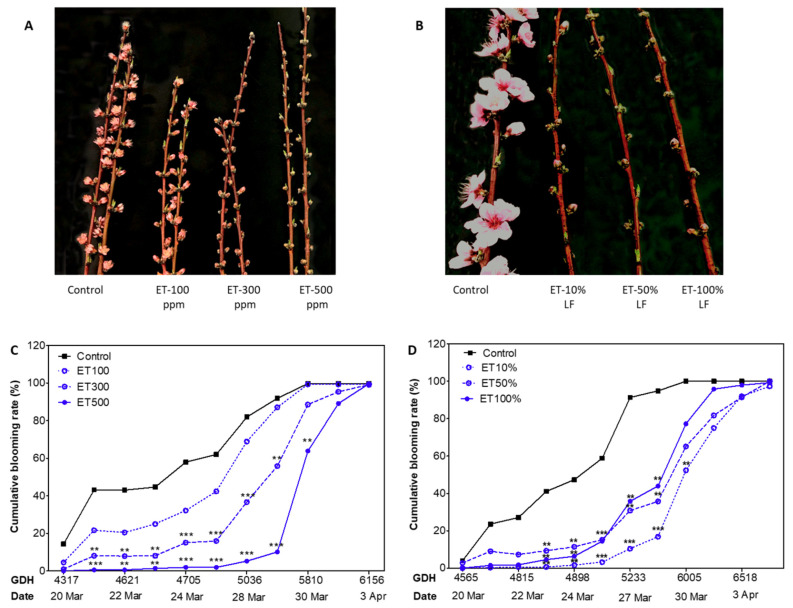
Bloom-delaying effects of ethephon (ET) on peach cultivars. Comparison of blooming between the untreated controls and ethephon treatments of ‘Redhaven’ (**A**) and ‘Sunhigh’ (**B**), with photos taken at full bloom of the controls (24 March 2020). Blooming progression of ‘Redhaven’ (**C**) and ‘Sunhigh’ (**D**) as affected by ethephon, and blooming rate calculated as the ratio of number of flowers to the number of buds on tagged branches. Each point represents the mean of size *n* = 12. Asterisks ** and *** indicate significant difference between ET treatments and the control at *p* < 0.01 and 0.001, respectively. ET, ethephon; LF, leaf fall.

In general, ethephon application induced remarkable bloom delay in peach in a concentration-timing and application-timing dependent manner (Figure 4A,B). In ‘Redhaven’, the flowering period lasted from mid-March to the beginning of April, with flowering date (F_50_) of control occurring on 23 March (Table 2). The three ethephon treatments resulted in progressive delays of the flowering date by 3–6 days, with the ET-300 and ET-500 ppm treatments showing significant bloom delays compared to the control (Figure 4C). Comparably, ‘Sunhigh’ exhibited 4–6 days delay in flowering date in all ethephon treatments (*p* < 0.05), with the ET-10% LF inducing the largest delay and ET-100% LF the lowest (Figure 4D).

### 3.7. Ethephon Effects on Fruit Set and Fruit Size

**Figure 5:**
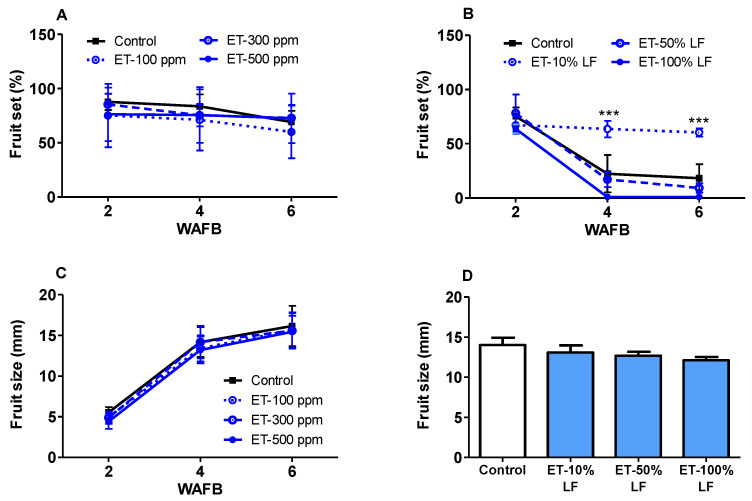
Ethephon effects on fruit set and fruit size of peach cultivars. Fruit set (%) of ‘Redhaven’ (**A**) and ‘Sunhigh’ (**B)** was evaluated 2, 4, and 6 WAFB. Fruit size of ‘Redhaven’ (**C**) was measured 2, 4, and 6 WAFB and fruit size of ‘Sunhigh’ (**D)** was measured 6 WAFB. Each point or bar represents mean ± SE, each of size *n* = 12 for fruit set, and *n* = 30 for fruit size. Asterisks *** represent significant difference between ET treatment and the control at *p* < 0.001. ET, ethephon; LF, leaf fall; WAFB, weeks after full bloom.

In this study, ethephon effects on percent fruit set and fruit size were evaluated 2, 4, and 6 weeks after full-bloom (WAFB). Fruits were thinned 7 WAFB and no further data of fruit set and fruit size were collected at harvest. In ‘Redhaven’, percent fruit set decreased gradually in all treatments and control, reaching 60–75% at 6 WAFB. No significant differences in the fruit set were observed between the control and ethephon treatments at any time point (Figure 5A). In ‘Sunhigh’, except for ET-10% LF treatment, a dramatic drop in fruit set was observed in the control and ET-50% and ET-90% treatments at 4 WAFB, which ranged from 70 to 98% (Figure 5B). Such abnormal fruitlet abscission could be presumably attributed to a spring frost that occurred in the ‘Sunhigh’ orchard between 2 and 3 WAFB on 17 April when the fruit size was about 5.5 mm. The lowest temperature of this frost reached −2.8 °C and lasted for more than 90 min (Figure S3).

The fruit size of ‘Redhaven’ and ‘Sunhigh’ was estimated by measuring the equatorial diameters of the tested fruits during the first 6 WAFB. In ‘Redhaven’, the fruit size of the control increased from 5.3 mm at 2 WAFB to 15.3 mm at 6 WAFB, with the ET-500 ppm treatment being 1.1 mm less than the control (*p* < 0.05) at 2 WAFB, and no difference between the control and the ethephon treatments found at 4 or 6 WAFB. In ‘Sunhigh’, the fruit size measured 6 WAFB was similar between the control and ethephon treatments, with the average diameter ranging 12.1 to 14.0 mm, showing a weak negative correlation between fruit size and earliness of ethephon application (Figure 5D).

### 3.8. Tree Injuries Associated with Ethephon 

**Figure 6:**
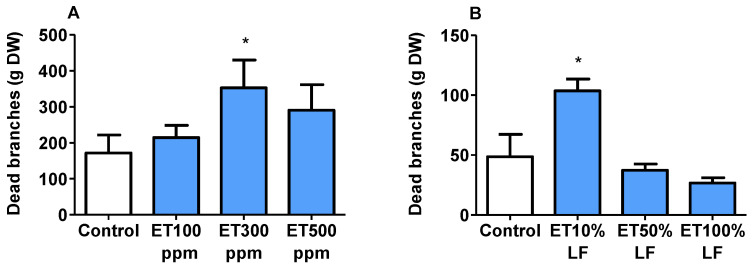
Ethephon effects on injury of peach ‘Redhaven’ (**A**) and ‘Sunhigh’ (**B**). Injury was evaluated by weighing the total dead branches from each tree 6 WAFB. Each bar represents the mean + SE of size *n* = 6. Asterisk * indicates significant difference between ET treatment and the control at *p* < 0.05. ET, ethephon; LF, leaf fall.

In this study, the weight (g) of dead branches collected 6 WAFB from each treated and control tree was used to estimate the magnitude of injury induced by ethephon. In ‘Redhaven’, the weight of dead branches per tree in control averaged at 171 g (± 50.1) and was significantly lower than that of the ET-300 ppm treatment (*p* = 0.039), but not different from ET-100 or ET-500 ppm (Figure 6A). In ‘Sunhigh’, the highest weight of dead branches was found in the ethephon 10% LF treatment (Figure 6B), averaging at 103.7 g (± 9.9) and was significantly higher than that of the control (*p* = 0.03). These results suggest that early applications of ethephon at high concentration (500 ppm) is likely damaging. 

### 3.9. Phytohormone Accumulation Profiles during Dormancy

Phytohormones play a critical role in regulating the processes of dormancy and flowering [29]. To test the hypothesis that fall-applied ethephon affects CR, HR, and bloom time through modulation of phytohormone biosynthesis and accumulation during dormancy, the levels of some major phytohormones were quantified in the floral buds throughout the bud dormancy cycle in relation to CH and GDH. In this study, we quantified the levels of phytohormones in the control and ET-500 ppm treatment of ‘Redhaven’ peach trees at 200, 400, 600, 800, and 1000 CH; and 1000 and 3000 GDH. 

**Figure 7:**
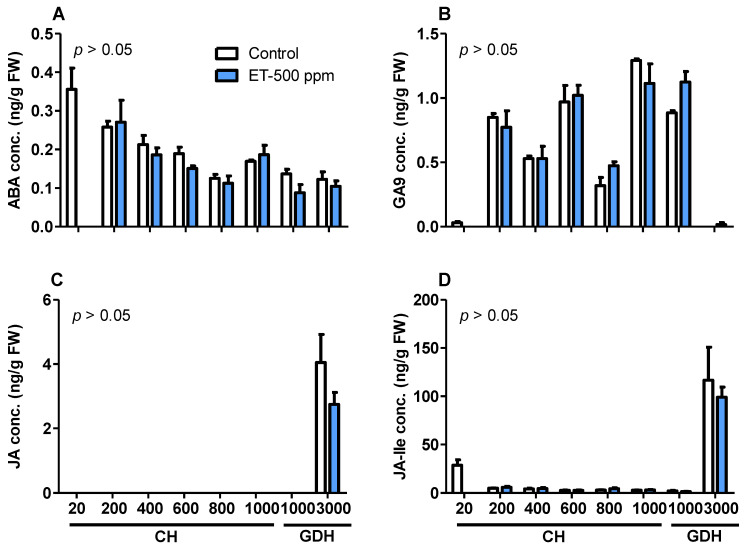
Hormone concentration of ABA (**A**), GA9 (**B**), JA (**C**), and JA-Ile (**D**) in floral buds of peach ‘Redhaven’ during endodormancy and ecodormancy. Each bar represents the mean ± SE of three biological replicates; *p*-values indicate comparisons between the control and ethephon treatment at 500 ppm. The CH (200, 600, 800, and 1000) and GDH (1000 and 3000) accumulation corresponds to the sampling dates of 11 November, 27 November, 9 December, and 27 December of 2019, and 15 January, 14 February, and 12 March of 2020, respectively. ABA, abscisic acid; GA, gibberellin; JA, jasmonic acid; and JA-Ile, Jasmonoyl isoleucine; CH, chilling hour; GDH, growing degree hour; conc., concentration; FW, fresh weight.

Of the four nongaseous major phytohormones, the levels of auxin, cytokinin, and all bioactive forms of gibberellin (GA) in the bud tissues were below the detectable level with the LC-MS system. The hormones successfully quantified were abscisic acid (ABA), GA9, a precursor of GA4, which is a bioactive gibberellin, and jasmonic acid (JA) and its conjugate Jasmonoyl-Isoleucine (JA-Ile). During the dormancy to the preflowering period, the changes in ABA levels were similar between the control and the ethephon treatments, with the highest level detected at 200 CH. ABA levels decreased gradually with chilling accumulation, reaching its lowest level at 800 CH and remained low until 3000 GDH, except for a moderate rise at 1000 CH (Figure 7A). Moderate fluctuations were observed in the levels of GA9 during dormancy, which reached its peak levels of 1.3 and 1.1 ng·g^−1^ in control and ethephon treatment at 1000 CH and declined slightly at 1000 GDH (Figure 7B). It was also noticeable that GA9 levels declined abruptly to nearly zero at 3000 GDH. In contrast to GA9, the levels of JA were undetectable from 200 CH through 1000 GDH, and abruptly increased to 4.1 and 2.8 ng·g^−1^ at 3000 GDH for the control and ethephon treatment, respectively (Figure 7C). Similarly, JA-Ile levels remained less than 6 ng·g^−1^ at all timepoints before 3000 GDH and spiked dramatically to 116.8 and 99.1 ng·g^−1^ at 3000 GDH for the control and ET treatment, respectively (Figure 7D). No significant differences were detected in the hormone levels between the control and ET treatment at any timepoint. 

### 3.10. Expression of Genes That Regulate ABA, GA, ET and JA Biosynthesis

**Figure 8:**
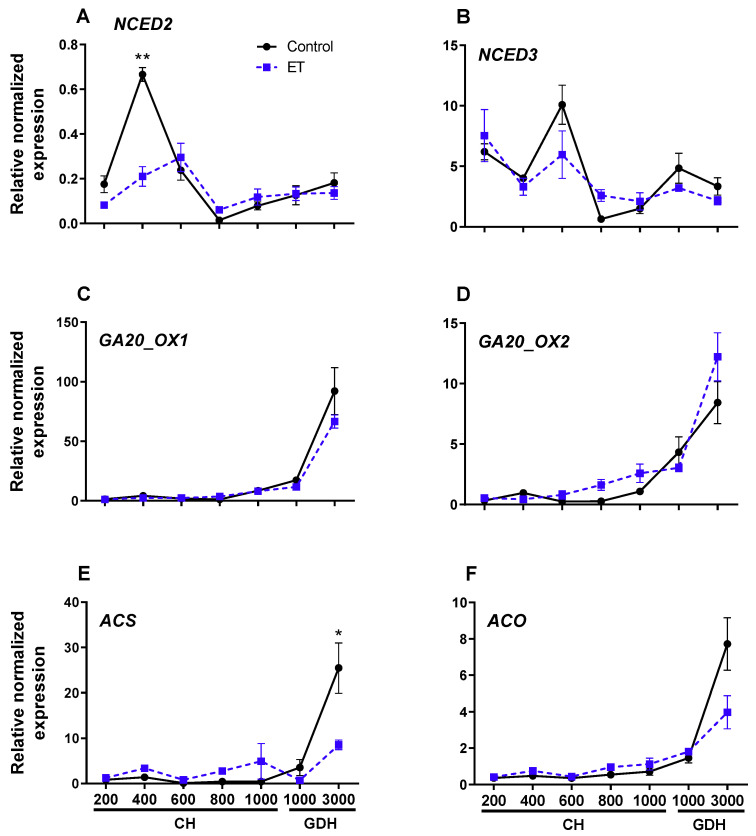
Relative normalized expression of genes that regulate biosynthesis of ABA (**A**,**B**), GA (**C**,**D**), and ethylene (**E**,**F**) in the floral buds of peach ‘Redhaven’. Expression of each gene was normalized to that of *β*-*Actin* and expressed relative to the control sample (20 CH). Each point represents the mean ± SE of three biological replicates, each with three technical replicates. Values marked with asterisks * and ** are significantly different at *p* < 0.05 and 0.01, respectively. NCED, 9-cis-epoxycarotenoid dioxygenase; GA20-OX, GA 20-oxidase; ACS, 1-aminocyclopropane-1-carboxylic acid (ACC)-synthase; and ACO, ACC-oxidase. CH, chilling hour; GDH, growing degree hour. Refer to Figure 7 legend for the sampling dates that correspond to the CH and GDH accumulation.

To further examine the effects of ethephon treatments on hormone de novo biosynthesis in the buds, the expression profiles of several key genes responsible for biosynthesis of GA, ABA, ET, and JA were investigated in ‘Redhaven’ in relation to CH and GDH. The results indicated that the expression of ABA synthetic gene *NCED2* was low at 200 CH for both the control and ethephon-500 ppm treatment, reaching its highest levels at 400 CH and 600 CH for control and ET treatment, respectively, followed by a rapid decline and remained relatively low until 3000 GDH (Figure 8A). Expression of *NCED3* was similar to *NCED2*, except for the highest expression of control and ET treatment both occurred at 600 CH (Figure 8B). For GA biosynthesis pathways, transcript levels of *GA20-OX1* and *GA20-OX2* showed low levels through the endodormancy period, and increased gradually with GHD accumulation, reaching a peak at 3000 GHD without showing significant differences between the control and ET treatment (Figure 8C,D). The two genes that regulate ethylene biosynthesis, *ACS* and *ACO*, showed similar expression patterns: both remained low from 200 CH through 1000 GDH and increased rapidly at 3000 GDH (Figure 8E,F). In particular, the ACS expression level in the control was two times higher than that of the ET treatment (*p* < 0.05). 

**Figure 9:**
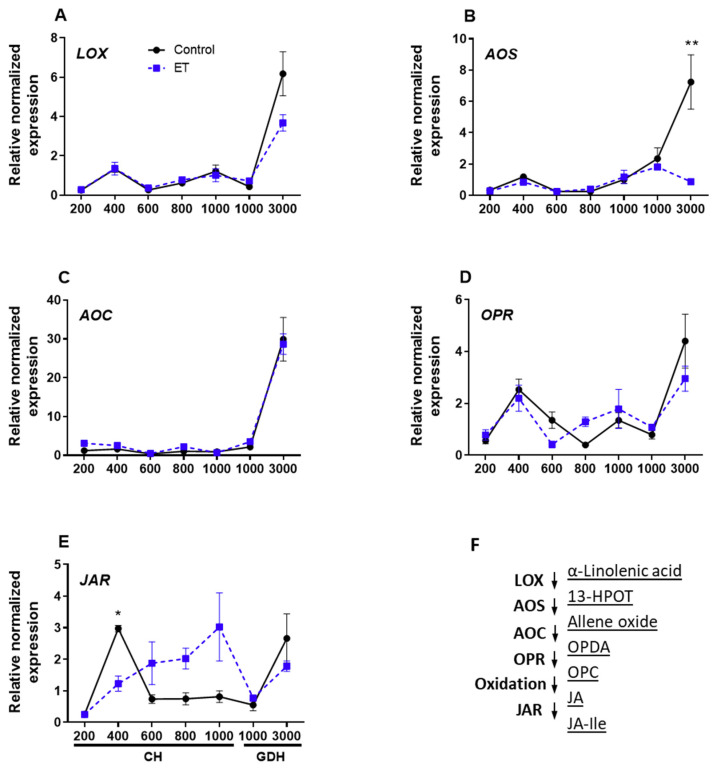
Relative normalized expression of JA synthetic genes in peach ‘Redhaven’ during bud dormancy (**A**–**E**) and the schematic diagram of the JA biosynthesis pathway (**F**). Expression of each gene was normalized to that of *β*-*Actin* and expressed relative to the control sample (20 CH). Each point represents the mean ± SE of three biological replicates, each with technical replicates. Values marked with asterisks * and ** are significantly different at *p* < 0.05 and 0.01, respectively. Enzymes that sequentially catalyze the synthesis of JA and conjugation of JA-Ile are listed on the left of the downward arrows, and intermediate products are given on the right (**F**). LOX, lipoxygenase; AOS, allene oxide synthase; AOC, allene oxide cyclase; OPR, OPDA reductase; JAR, JA-amino synthetase; OPC, cyclopentane-1-octanoic acid; OPDA: oxo-phytodienoic acid; JAR, JA-amino acid synthetase. CH, chilling hour; GDH, growing degree hour. Refer to Figure 7 legend for the sampling dates that correspond to the CH and GDH accumulation.

To unravel the transcriptional regulation of JA and JA-Ile during dormancy, we examined the transcriptional activity of the genes that control the key steps in the biosynthesis JA and JA-Ile. JA biosynthesis starts from an 18-carbon fatty acid (α-linolenic acid, 18:3), which undergoes multistep oxygenation, cyclization, and reduction that are sequentially catalyzed by linoleate 13S-lipoxygenase (13-LOX), allene oxide synthase (AOS), allene oxide cyclase (AOC), and OPDA-reductase 3 (OPR3); in the final step, JA is converted to the most bioactive form jasmonoyl-isoleucine (JA-Ile) by JA-amino synthetase (JAR) [30] (Figure 9F). Our results indicated that the relative expression of *LOX* (Figure 9A) remained low from 200 CH through 1000 GDH and increased rapidly at 3000 GDH to 6.2 and 3.7 for control and ET treatment, respectively, with no significant difference between the two. The expression of *AOS* (Figure 9B) in the control and ET treatment were both low at 200 CH and through 1000 GDH. At 3000 GDH, *AOS* transcript levels in the control increased abruptly, whereas the expression of the ethephon treatment decreased slightly, being about 10% of the control (*p* < 0.01). Nearly identical transcript dynamics of *AOC* were found between the control and ethephon treatment throughout all the timepoints (Figure 9C), in which both remained extremely low until 3000 GDH, when both exhibited an abrupt increase. Similar expression pattern was also found for *OPR3* (Figure 9D), in which the expression increased only slightly at 3000 GDH. The expression of *JAR* (Figure 9E) appears to lack a clear pattern. *JAR* transcripts in the control remained low from 200 CH to 1000 GDH, except for a noticeable increase at 400 CH, which was higher than the ET treatment (*p* < 0.01), and another clear peak at 3000 GDH. In the ET treatment, *JAR* transcript levels were slightly higher than that of the control during dormancy period, but the differences were not statistically significant.

## 4. Discussion

Our study extensively examined the ethephon effects on several key physiological aspects during the dormancy-bloom period of two peach cultivars. Through field trials and laboratory experiments, we showed that fall applications of ethephon significantly modulated chilling and heat requirements, acclimation/deacclimation of dormant buds, cold hardiness, bloom date, and overall tree health. Hormonal quantification of ethephon-treated and untreated trees revealed that JA biosynthesis pathway may play an essential role in triggering budburst and flowering in peach, and gene expression profiles also confirmed that de novo JA biosynthesis in the buds could be also affected by the ethephon treatment. 

### 4.1. Floral Bud Phenology and Cold Hardiness as Modulated by Ethephon 

Chilling and heat requirements have been shown to be essential factors that dictate flowering date in deciduous woody perennials, although some disagreement exists regarding their relative importance [31,32,33,34,35]. Ethephon effects on increasing cold and heat requirements have been reported in several studies. Using container-grown peach trees, Durner and Gianfagna [11] showed that ‘Redhaven’, when treated with 100 ppm ethephon, required three more weeks of chilling exposure to release from endodormancy compared to the control. Similarly, our results indicate ethephon extended both CR and HR in peach, in a concentration and application time-dependent manner. Since chilling requirements were largely fulfilled before the onset of heat accumulation in all treatments, the extent by which ethephon affected bloom delay should only be explained by the increased HR. 

In the ethylene production experiment, our results showed that application of ethephon induced rapid increase of ethylene in leaves, confirming the notion that leaves are the primary organs that absorb and convert ethephon [27,28]. This was further confirmed in flower buds where ethylene levels were significantly higher when ethephon was applied at 10% compared to 90% LF, asserting that leaves are required for effective uptake of ethephon that is subsequently converted and distributed to other organs. Notably, ethephon applied at 10% LF stimulated an ethylene spike in the flower buds two days after application, followed by a rapid decrease. This result is consistent with a recent study [36] in which ethylene evolution in peach flowers and fruitlets exhibited similar dynamics in response to foliar application of ethephon, which induces fruit abscission. Likely, it is this transient increase of ethylene in the flower buds that exert effects on the subsequent phenological and physiological processes. 

Our results showed that ethephon treatment significantly delayed bloom date, and the extent of the delay depended on concentrations and time of application. Essentially, ethephon was more effective when applied at the early stage of leaf fall and at higher concentrations (Figure 3). Our results agree with several other reports that have shown varying effects of ethephon on bloom delay in response to different concentrations and application timings. For instance, Coston and Krewer [37] reported that 120 ppm ethephon delayed bloom in peach by 5 and 9 days, whereas 500 ppm ethephon delayed bloom by up to 18 days. In another study, fall applications of ethephon at 50, 200, and 400 ppm delayed the full bloom date of nectarine by 6–15 days [38]. The reason why fall-applied ethephon delays bloom is still unclear. Some reports indicated that ethephon applications can retard the growth of flower buds and floral organs [37], and this delayed growth could be linked to the increase of ABA levels in the dormant buds induced by ethephon [39]. Indeed, ethylene has been shown to promote the synthesis of ABA [40,41] and to activate ABA signaling pathway [42] as well. However, our results, cannot support this ethephon-induced ABA hypothesis as we did not observe any significant effects for ethephon on the levels of ABA and its biosynthetic genes at any time point during the bud dormancy of ‘Redhaven’ peach (Figure 7A and Figure 9A,B). 

Recently, Liu and Sherif [4] implied that the ethephon-induced bloom delay could be a result of elevated stress response. If exogenous ethephon application is to be sensed by the plant as a stress signal, the extended dormancy period and delayed bloom would be advantageous for the fitness and survival for the perennial species in face of unfavorable environmental conditions [43]. There is emerging evidence that supports this hypothesis. Gummosis is the exudation of polysaccharide gum that is produced primarily in response to various stresses [44], and ethephon has been implicated in the induction of gummosis in stone fruits [45]. In addition, the treatment with ethylene immediate precursor 1-aminocyclopropane-1-carboxylic acid (ACC) has been found to trigger cell cycle arrest in *Arabidopsis* [46] and leafy spurge [42], in a way comparable to stress responses. In fact, growth arrest is a prerequisite for the initiation of dormancy, and hastened growth cessation has been associated with intensified dormancy and delayed bud burst [47]. It is thus likely that fall-applied ethephon stimulates an array of stress responses, which are retained throughout the dormancy period until the budburst and flowering. 

Flower buds contain reproductive primordia, and their ability to withstand the freezing temperatures during winter and spring determines the yield potential and fruit quality. In this study, the cold hardiness of ‘Redhaven’ flower buds was examined in control and the 500 ppm ethephon treatment during dormancy and through the prebloom stage. The steady rise of hardiness occurred approximately when the CR was fulfilled in control (1046 CH) and the ethephon treatment (1177 CH). This loss of cold hardiness, or deacclimation is a function of many external and internal factors, such as ambient temperatures, photoperiod, water availability, metabolism status, and dormancy status [48]. Change in cold hardiness in peach is closely related to the soluble sugar content and the accumulation of a 60 kDa dehydrin protein [49]. Our results indicate that cold hardiness is highly dependent on the depth of dormancy and was improved by ethephon applications (Figure 2B), which is generally consistent with other reports on grapevine [50], peach [6], and magnolia (*Magnolia wufengensis*) [51]. Peach buds acquire cold hardiness primarily via the supercooling of intracellular water, and it was shown that ethephon enhances supercooling in peach buds through increasing soluble sugar content [52]. Ethylene has been implicated in the development of chilling tolerance. With an ethylene-insensitive mutant of tomato (*Lycopersicon esculentum*), [53] demonstrated that chilling tolerance can be enhanced by increased ethylene biosynthesis. The mechanism by which ethephon enhances the cold tolerance in plants has also been implied in the activation of ethylene response factor (*ERF*) genes, which activate several cold-response pathways, including antioxidation, ice nucleation activity, and production of osmotic proteins [54,55]. In *Magnolia* shoots, ethephon was shown to reduce the water content and facilitate the accumulation of free proline [51], a beneficial amino acid that imparts stress tolerance, including cold [56,57].

### 4.2. Ethephon Effects on Fruit Set, Fruit Size and Tree Health

Strong fruit set is critical for orchard profitability and sustainability. In this study, the fruit set was not significantly influenced by ethephon in ‘Redhaven’, when applied at concentrations up to 500 ppm (Figure 5A and Figure 6B). In ‘Sunhigh’, as the fruit set was dramatically reduced in control along with the 50 and 100% LF treatments, only slight reduction was observed in the in 10% LF treatment. This remarkable decline in fruit set (%) in the ‘Sunhigh’ orchard was likely due to the spring frost that occurred 2 WAFB (17 April 2020), which reached −2.8 °C and lasted for 90 min (Figure S3). Such frosts were not recorded in the ‘Redhaven’ orchard, probably because of its higher elevation (7 m higher) compared to the ‘Sunhigh’ orchard, making it less prone to cold air accumulation at night. The critical temperature that kills 10 and 90% of post bloom flowers within 30 min are −2.2 and −3.9 °C, respectively (https://www.canr.msu.edu/resources/picture-table-critical-spring-temperatures-for-tree-fruit-bud-development-stages, accessed on 15 May 2021). Therefore, the frosts that inflicted the ‘Sunhigh’ orchard were severe enough to damage or kill the developing fruitlets that averaged at 5.5 mm in diameter. The unaffected fruit set (%) found in ethephon treatment at 10% LF should reflect the improvement of cold hardiness in this treatment. The higher effectiveness of ethephon applied at 10% LF than later stages is likely because leaves are the primary site where ethephon is absorbed and quickly hydrolyzed to release ethylene after entering the leaf cells [58], and more leaves apparently accelerate this process. 

The final fruit size at harvest is predominantly determined by the cell number, whereas cell size only has minor effects [59]. In this study, the fruit size of ‘Redhaven’ and ‘Sunhigh’ was measured in the first six weeks after bloom (42 DAB), at which the differences of fruit size between treatments and the control would adequately reflect the relative fruit size at harvest. Our results showed there was no significant difference between the ethephon treatments and the control in either orchard. This finding is similar to the observation by Irving [38] that ethephon had no adverse effects on fruit size of nectarine at harvest. In contrast, some studies indicated that fall-applied ethephon causes reduction in the fruit size in peach [60] and sweet cherry. The final fruit size is highly dependent on many factors such as fruit cell number/size, pre-thinning/post-thinning crop load, rate of fruit development, and those factors that may potentially confound with the use of ethephon, such as spring frosts and ethephon-induced injury. The discrepancies in the findings may arise from any of these factors, and accurate examination of fruit size will require these confounding factors to be kept constant or eliminated. 

Tree injury is one of the common issues that has been linked to ethephon applications [10,37]. Our results indicated that both ethephon concentration and application timing are important factors that contribute to the damaging impact of ethephon, as more damage to tree branches was observed with high concentrations and early stage of leaf fall (Figure 6B). Since ethephon enters plants through the leaf epidermis, applications at early stage of LF, when more leaves are still attached, would elicit higher ethylene response. Branch damage is only one aspect of the ethephon-related injury, and whether such damage would affect cumulative tree yield over several years warrants further investigation. 

### 4.3. Endogenous Hormonal Changes Associated with Bloom Delay 

ABA has been regarded as the fundamental cornerstone in the regulatory network of bud dormancy. In this study, ABA levels was high at the beginning of endodormancy, and gradually decreased thereafter with the accumulation of chilling units (Figure 7). This result supports the notion that ABA is responsible for dormancy induction and is consistent with observations reported in grapevine [61], peach [62], and pear (*Pyrus pyrifolia*) [63]. ABA accumulation pattern during dormancy cycle was also supported by the transcript levels of ABA biosynthesis genes, *NCED2* and *NCED3* (Figure 8). However, it can be noted that expression level of these genes somehow lagged behind the changes in ABA levels. This discrepancy between ABA increase and *NCED* transcriptional induction was also observed in grapevine [61], which suggests that high ABA levels may be induced by *NCED* paralogs other than *NCED2* and *NCED3* at the endodormancy initiation. It is also worth noting that ABA’s role as dormancy mediator is not necessarily involved in the ethephon-mediated bloom delay. In fact, the level of ABA and its biosynthetic genes showed no significant difference between the control and the ethephon-treated trees at any time point. This result indicates that ethephon may exert its effects on CR, HR, and bloom delay through mechanisms that are independent of ABA. 

Gibberellins (GA) comprise a large group of diterpenoid tetracyclic acids, in which only a small subset possesses bioactivity. In GA biosynthesis, GA9 is synthesized from GA12 by a series of oxidation catalyzed by GA20-oxidase (GA20ox) followed by oxidation by GA3-oxidase (GA3OX) to from the bioactive GA4. It has been shown that GA4 plays a role in the regulation of *Arabidopsis* floral initiation through modulating *LFY* transcription [64]. Application of GA4 has been shown to induce bud burst in *Populus* [65] and bud dormancy release in Japanese apricot [66]. It is unexpected that of the many forms of GA, only the level of GA9 was detectable (Figure 7B). GA9 level was relatively high during endodormancy and ecodormancy, and decreased dramatically at 3000 GDH. As an inactive form, GA9 may serve as a GA reserve during dormancy when the active forms of GA are not in need. The rapid decrease of GA9 at 3000 GDH, therefore, could reflect the rapid oxidation of GA9 to form GA4, which in turn, triggers bud burst and flowering. Further investigation is needed to validate this hypothesis. The upregulation of *GA20ox* at 3000 GDH suggests the de novo synthesis of GA in floral buds (Figure 8C,D). Indeed, the increase of *GA20ox3* expression was concomitant with the release of endodormancy in grapevine [67]. 

JA has complex effects in flower opening and development of floral organs [68]. As the inhibitory effect of JA on flowering was found in morning glory (*Pharbitis nil*) [69] and *Arabidopsis* [68], its promotive effect was showed in tomato (*Solanum lycopersicum*) [70]. In addition to its functions on flowering, JA has also been implicated in dormancy release of seeds, which is exemplified by a transient increase of JA preceding germination [71,72]. Direct evidence of JA’s role in bud dormancy is scarcely documented. In analyzing the transcriptome changes in *Populus*, Howe et al. [73] exhibited that several genes that regulate JA synthesis were downregulated at the onset of dormancy, whereas two *LOX* genes were upregulated at the release of dormancy. JA gains its hormonal activity via conjugation, catalyzed by JAR, with isoleucine to form JA-isoleucine (JA-Ile) [74]. In the present study, the levels of JA and JA-Ile peaked 7–10 days before the initial flowering date in both the control and ethephon-treated trees (Figure 7C,D), indicating that JA may play a role in inducing bud burst and bloom. The gene expression analysis revealed that the expression of several genes regulating JA synthesis such as *LOX*, *AOS*, *AOC,* and *OPR3* were all upregulated concomitant with the rise of endogenous JA levels prior to flowering (Figure 9A–D), indicating that JA may be de novo synthesized and regulated transcriptionally in the buds. This increase of JA and JA-Ile levels prior to flowering is in line with the finding by Ionescu et al. [75], who demonstrated that JA and JA-Ile levels in sweet cherry increased remarkably before budburst, along with the upregulation of several JA biosynthesis genes. The authors also found that JA-Ile production was induced by the treatment of hydrogen cyanide, an agrochemical that is used commercially to induce budbreak in many deciduous fruit trees. It is also important to note that, the JA and JA-Ile levels in the ethephon treatment were 47.3 and 17.8% lower than those in the control, respectively (Figure 7C,D), but the differences were not statistically significant. However, this result may still be indicative of reduction of JA synthesis in ethephon treatments, as the expression of *AOS* in the ethephon treatment was only 10% of that of the control (Figure 9B), and AOS controls a key step in JA biosynthesis [76]. 

Some molecular evidence has shown that both ethylene biosynthesis and signaling are involved in the induction of dormancy. Treatment with the ethylene antagonist, 2,5-norbornadiene (NBD), promotes dormancy release in potato microtuber [77], and impairment in ethylene perception inhibits the dormancy initiation in chrysanthemum (*Chrysanthemum morifolium*), even after treated with ethephon [78]. The finding that a suite of ethylene signaling genes (e.g., *ETR2*, *EIN3*, *EIN4*, and *ERFs*) in *Populus* is upregulated under dormancy-inducing conditions [79] reinforces the role of ethylene in dormancy establishment. In higher plants, ACC synthase (ACS) and ACC oxidase (ACO) are the two pacesetting enzymes in ethylene biosynthesis, with their expression stringently controlled [80]. In this study, the upregulation of ACS and ACO at 3000 GDH indicate an increase in ethylene biosynthesis (Figure 8E,F), which agrees with the notion that ethylene plays an important role in dormancy release and flowering [29,75,81]. It can be noted the ACS and ACO transcript levels were lower in the ethephon treatment than the control, suggesting that ethephon treatment in the fall may result in an eventual reduction in ethylene synthesis toward the end of ecodormancy, thereby inducing bloom delay. 

## 5. Conclusions

In this study, we demonstrated that fall application of ethephon impacted several aspects of peach floral bud phenology, including accelerated leaf fall, extended chilling accumulation period, increased heat requirements, improved cold hardiness, and delayed bloom date. In general, ethephon effects on these aspects depended on the concentration and application timing, with high concentrations and early fall applications being more effective. However, such application regimes may be coupled with damages to peach trees and potentially negate the beneficial effects of ethephon in bloom delay, which could subsequently restrain its commercial use for frost mitigation. The hormonal and molecular data collected in this study presented evidence that ethephon-exerted effects on floral bud phenology are unlikely reliant on ABA and its biosynthetic pathway. Therefore, it is rather unlikely that ABA would serve as a plausible ethephon alternative to delay bloom in stone fruits. On the other hand, our data signifies the role of JA as a potential regulator of budburst and bloom in peach; however, further research is required to validate these observations and investigate the underlying crosstalk between JA, ethylene, and other dormancy-related pathways. Our findings portray a new perspective in interpreting horticultural traits in the light of biochemical and molecular data, and explain, at least partially, the role of ethylene in dormancy and flowering phenology. This research also reinforces the use of CH and GDH as universally applicable markers to schedule sample collections and record phenological events. Using these markers in future studies will enable parallel and comparative analyses of physiological and omics data to fine-tune the inherently complex pathways governing bud dormancy in peach and other deciduous woody perennials. Ethephon-mediated bloom delay could serve as a useful model for investigating the genetic, epigenetic, and biochemical mechanisms underlying flowering time regulation in peach. Understanding these aspects is not only essential for developing climate-resilient germplasm through breeding and biotechnology, but it can also help in formulating products and strategies to modulate the bloom dates of existing cultivars, hence avoiding potential spring freezes. 

## Figures and Tables

**Table 1:** Chilling requirements of peach cultivars as affected by autumn-applied ethephon.

Cultivar	Treatment	CR ^x^	Date ^y^	Days ^z^	
Redhaven (*P. persica*)	Control	1046 (62.2)	22 January	88	a
	ET-100 ppm	1112 (65.9)	26 January	92	ab
	ET-300 ppm	1147 (67.3)	28 January	94	ab
	ET-500 ppm	1177 (67.8)	29 January	95	b
Sunhigh (*P. persica*)	Control	805 (46.9)	28 December	94	a
	ET-10%	1145 (68.4)	27 January	124	b
	ET-50%	1115 (65.6)	26 January	123	b
	ET-100%	1092 (63.1)	25 January	122	b

^X^ Chilling requirements (CR) is displayed as the number of CH and CP (in parenthesis). ^Y^ Date at which 50% of buds released from endodormancy. ^z^ Number of days from the beginning of chilling accumulation (7 October) until release of endodormancy. All comparisons are made within each cultivar with significant level at 0.05, and significant differences apply to both CH and Days.

**Table 2:** Heat requirements and flowering date of two peach cultivars as affected by autumn-applied ethephon.

Cultivar	Treatment	HR ^X^	Flowering (F_50_) ^y^	Days ^z^	
Redhaven (*P. persica*)	Control	4682	23 March	60	a
	ET-100 ppm	4927	26 March	63	ab
	ET-300 ppm	5260	28 March	65	b
	ET-500 ppm	5583	29 March	66	b
Sunhigh (*P. persica*)	Control	4960	24 March	61	a
	ET 10%	5842	30 March	67	b
	ET 50%	5656	29 March	66	b
	ET 100%	5543	28 March	65	b

^X^ Heat requirements (HR) is displayed as the number of growing degree hours (GDH). ^Y^ Date at which 50% of buds were at the open-blossom stage (F_50_). ^z^ Number of days after breaking of endodormancy until flowering (F50). All comparisons are made within each cultivar with significant level at 0.05, and significant differences apply to both CH and Days.

## Data Availability

The data presented in this study are available in the supplementary materials.

## Supplementary Materials

The following are available online at https://www.mdpi.com/article/10.3390/plants10071266/s1, Figure S1. Evaluation of ethephon-induced injury on peach trees; Figure S2. Low Temperature Exotherm (LTE) analysis of cold hardiness; Figure S3. Temperatures 2 weeks after full bloom in the orchards of ‘Redhaven’ and ‘Sunhigh’; Table S1. Gene-specific primer sequences used for relative gene expression analysis of peach ‘Redhaven’ using qPCR.
