# Supplementary material for: Ethylene-Mediated Modulation of Bud Phenology, Cold Hardiness, and Hormone Biosynthesis in Peach (Prunus persica)
Source: Plants (Basel). 2021 Jun 22;10(7):1266. doi: 10.3390/plants10071266 (PMC8309013; doi:10.3390/plants10071266)
Supplement: Supplementary file 1 [file plants-10-01266-s001.zip › plants-1234882-supplementary.pptx]

## Slide 1
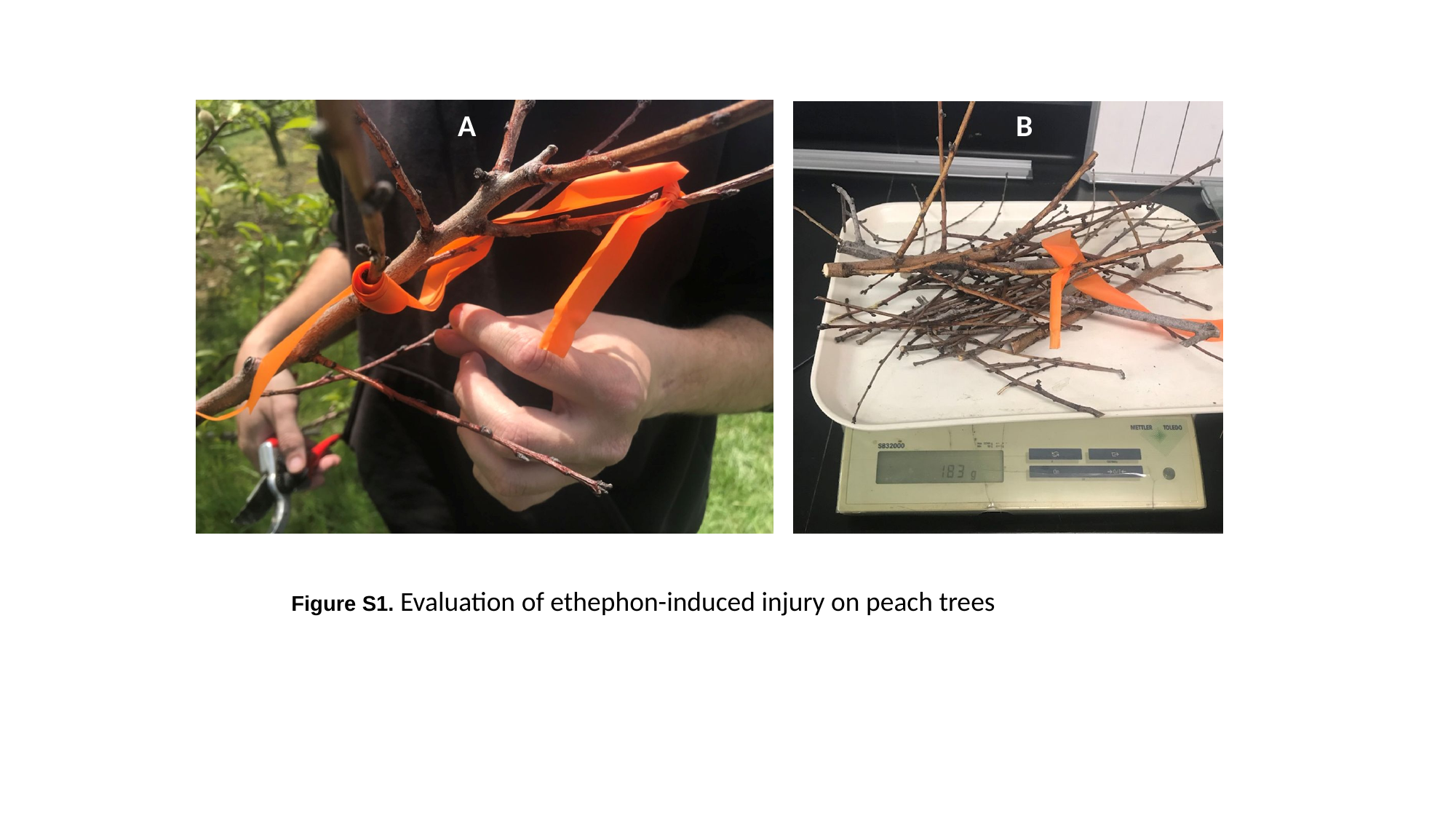

A
B
Figure S1. Evaluation of ethephon-induced injury on peach trees

## Slide 2
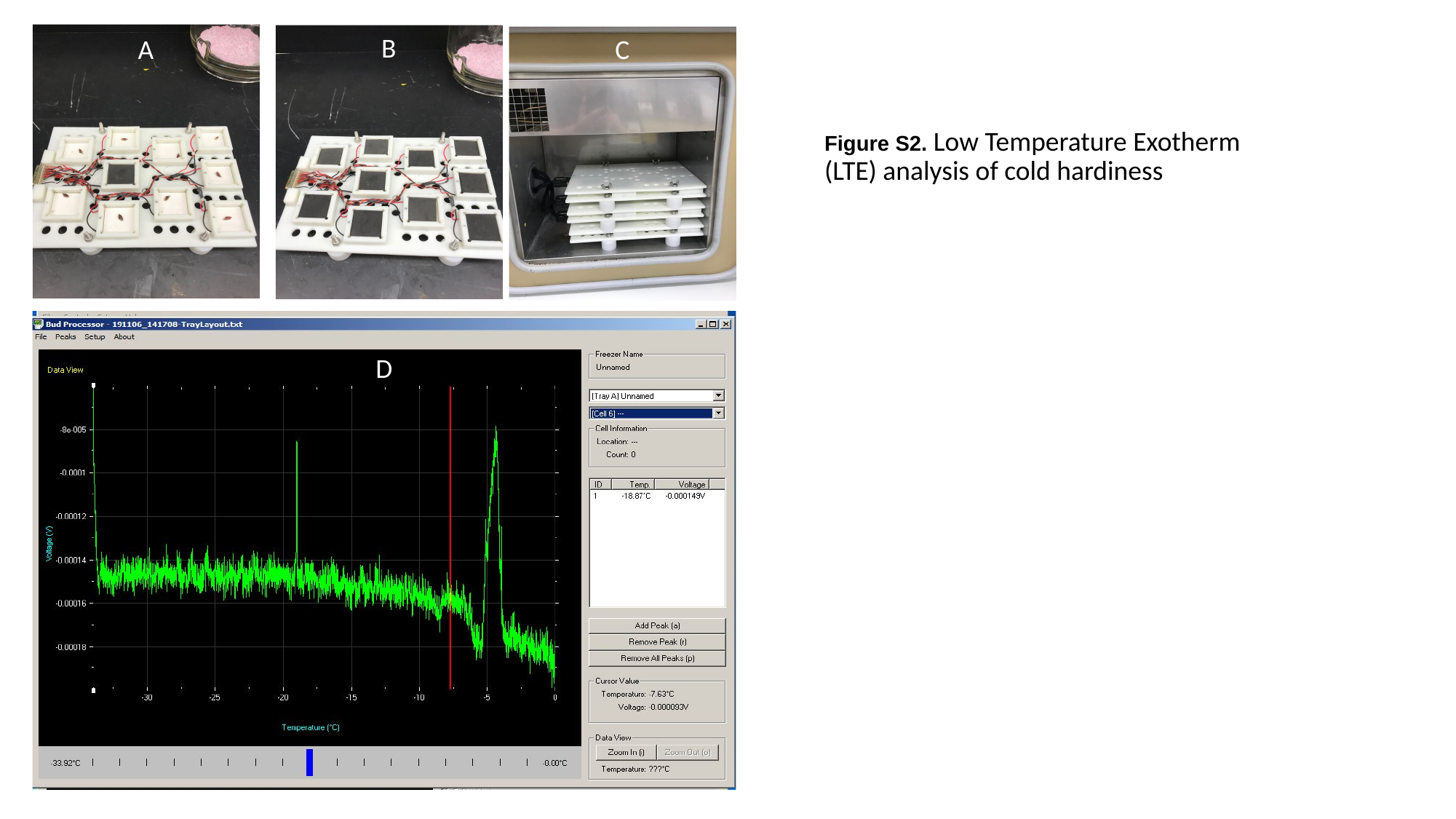

B
A
C
D
Figure S2. Low Temperature Exotherm (LTE) analysis of cold hardiness

## Slide 3
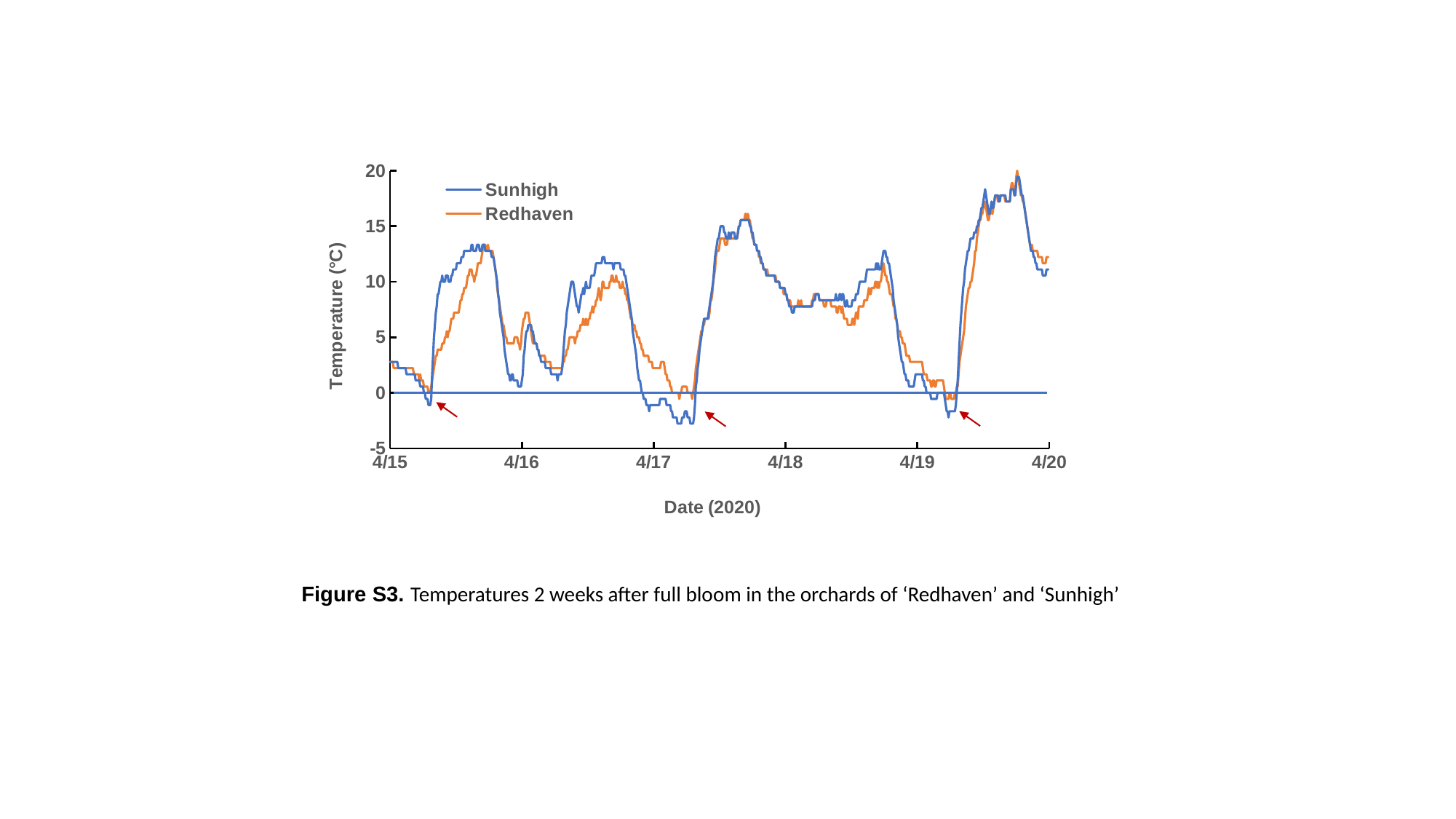

Figure S3. Temperatures 2 weeks after full bloom in the orchards of ‘Redhaven’ and ‘Sunhigh’
### Chart
| Category | Sunhigh | Redhaven |
|---|---|---|

## Slide 4
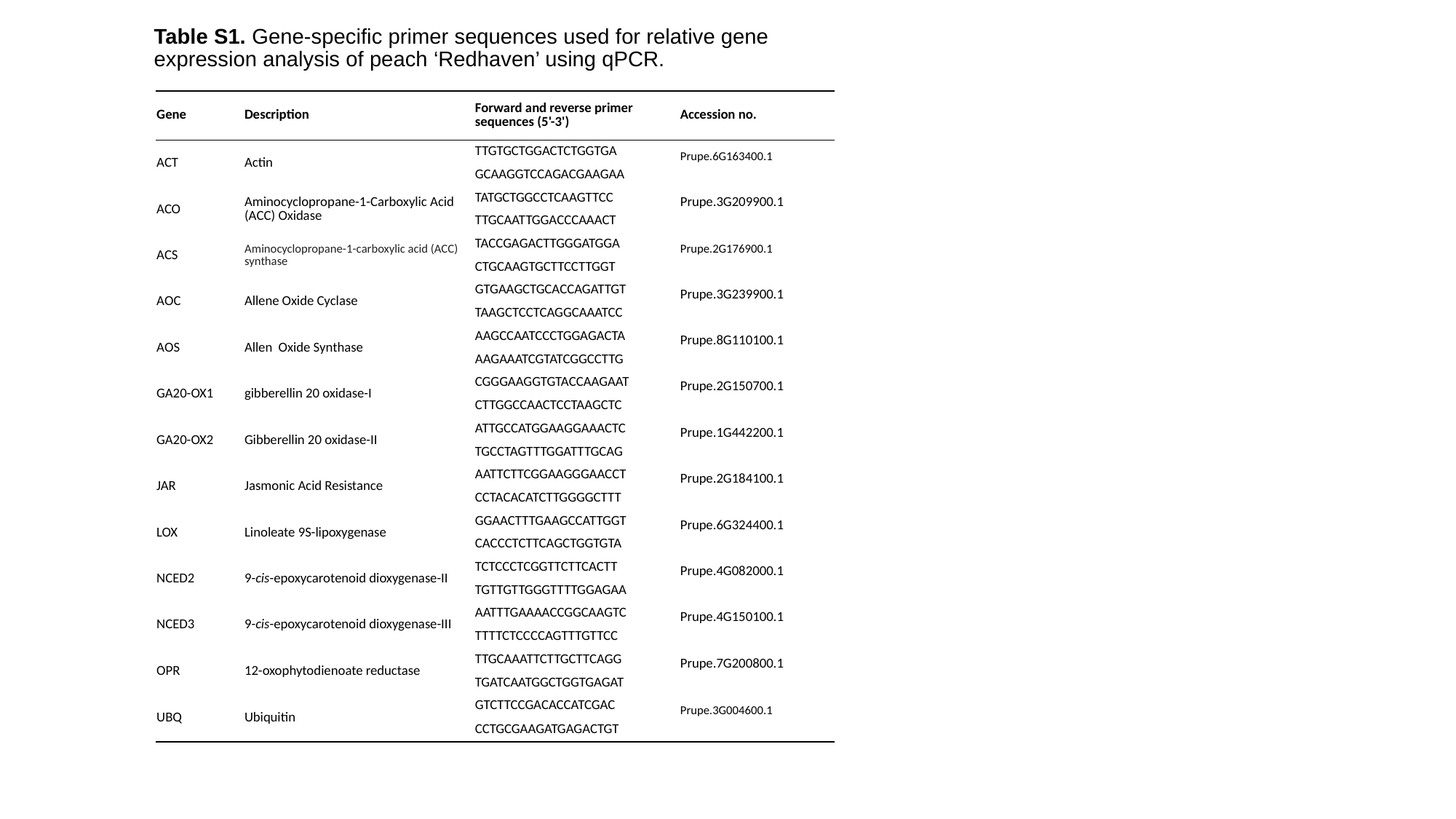

Table S1. Gene-specific primer sequences used for relative gene expression analysis of peach ‘Redhaven’ using qPCR.
| | | | | | |
| --- | --- | --- | --- | --- | --- |
| | Gene | Description | Forward and reverse primer sequences (5'-3') | Accession no. | |
| | ACT | Actin | TTGTGCTGGACTCTGGTGA | Prupe.6G163400.1 | |
| | | | GCAAGGTCCAGACGAAGAA | | |
| | ACO | Aminocyclopropane-1-Carboxylic Acid (ACC) Oxidase | TATGCTGGCCTCAAGTTCC | Prupe.3G209900.1 | |
| | | | TTGCAATTGGACCCAAACT | | |
| | ACS | Aminocyclopropane-1-carboxylic acid (ACC) synthase | TACCGAGACTTGGGATGGA | Prupe.2G176900.1 | |
| | | | CTGCAAGTGCTTCCTTGGT | | |
| | AOC | Allene Oxide Cyclase | GTGAAGCTGCACCAGATTGT | Prupe.3G239900.1 | |
| | | | TAAGCTCCTCAGGCAAATCC | | |
| | AOS | Allen Oxide Synthase | AAGCCAATCCCTGGAGACTA | Prupe.8G110100.1 | |
| | | | AAGAAATCGTATCGGCCTTG | | |
| | GA20-OX1 | gibberellin 20 oxidase-I | CGGGAAGGTGTACCAAGAAT | Prupe.2G150700.1 | |
| | | | CTTGGCCAACTCCTAAGCTC | | |
| | GA20-OX2 | Gibberellin 20 oxidase-II | ATTGCCATGGAAGGAAACTC | Prupe.1G442200.1 | |
| | | | TGCCTAGTTTGGATTTGCAG | | |
| | JAR | Jasmonic Acid Resistance | AATTCTTCGGAAGGGAACCT | Prupe.2G184100.1 | |
| | | | CCTACACATCTTGGGGCTTT | | |
| | LOX | Linoleate 9S-lipoxygenase | GGAACTTTGAAGCCATTGGT | Prupe.6G324400.1 | |
| | | | CACCCTCTTCAGCTGGTGTA | | |
| | NCED2 | 9-cis-epoxycarotenoid dioxygenase-II | TCTCCCTCGGTTCTTCACTT | Prupe.4G082000.1 | |
| | | | TGTTGTTGGGTTTTGGAGAA | | |
| | NCED3 | 9-cis-epoxycarotenoid dioxygenase-III | AATTTGAAAACCGGCAAGTC | Prupe.4G150100.1 | |
| | | | TTTTCTCCCCAGTTTGTTCC | | |
| | OPR | 12-oxophytodienoate reductase | TTGCAAATTCTTGCTTCAGG | Prupe.7G200800.1 | |
| | | | TGATCAATGGCTGGTGAGAT | | |
| | UBQ | Ubiquitin | GTCTTCCGACACCATCGAC | Prupe.3G004600.1 | |
| | | | CCTGCGAAGATGAGACTGT | | |
| | | | | | |
